# Three questions to ask before using model outputs for decision support

**DOI:** 10.1038/s41467-020-17785-2

**Published:** 2020-09-30

**Authors:** Volker Grimm, Alice S. A. Johnston, H.-H. Thulke, V. E. Forbes, P. Thorbek

**Affiliations:** 1 Helmholtz Centre for Environmental Research – UFZ, Department of Ecological Modelling, Permoserstr. 15, 04318 Leipzig, Germany; 2 University of Potsdam, Institute for Biochemistry and Biology, Maulbeerallee 2, 14469 Potsdam, Germany; 3 Cranfield University, School of Water, Energy and Environment, Bedfordshire, MK43 0AL UK; 4 Department of Ecology, Evolution and Behavior, University of Minnesota, 123 Snyder Hall, 1475 Gortner Avenue, St. Paul, MN USA; 5 BASF SE, APD/EE, Speyerer Straße 2, 67117 Limburgerhof, Germany

**Keywords:** Biological models, Ecological modelling, Infectious diseases, Scientific community

## Abstract

Decision makers must have sufficient confidence in models if they are to influence their decisions. We propose three screening questions to critically evaluate models with respect to their purpose, organization, and evidence. They enable a more transparent, robust, and secure use of model outputs.

Models are increasingly used to support decisions across environmental, economic, social, and public health issues. They deliver insights and possible solutions to real-world problems and allow decision makers to evaluate the consequences of their decisions before implementation. Examples include simulations of financial markets, fisheries, climate, and the spread of infectious diseases. On the one hand, models have helped make effective decisions, for example, in the eradication of rabies^1,2^. On the other hand, models have sometimes been trusted with disastrous consequences^3^ such as the collapse of the Atlantic cod fishery and the 2008 financial crisis.

As the world faces the uncertainty of the Covid-19 pandemic, models represent the most effective tool to identify interventions that can balance the risks of widespread infection and social disruption until an effective treatment is established^4^. Contradictory outputs from numerous models, however, reflect widely differing assumptions and purposes^5^. Divergent model outputs are not only confusing but underscore the need for clear communication of models and their context, so that decision makers can select the most appropriate models for the problem at hand.

Model communication has been previously addressed within the context of good modeling practice (GMP^6–8^). GMP, however, does not necessarily reflect the perspective of decision makers who at some point need to trust models and their output if they are to influence their decisions^9^. Decision makers want evidence that a model works, which relies on demonstrating the model’s realism and the reliability of data inputs and key assumptions. Here, we integrate central principles from GMP with the decision makers’ perspective to propose three screening questions (Fig. 1). The questions provide an overview of a model’s purpose, the assumptions underlying its organization, and the evidence that it is realistic enough for its purpose. Based on the ODD protocol for communicating models^10^, these questions should be addressed by modelers during model development, and by decision makers before a model’s outputs are used for decision support.

**Fig. 1:**
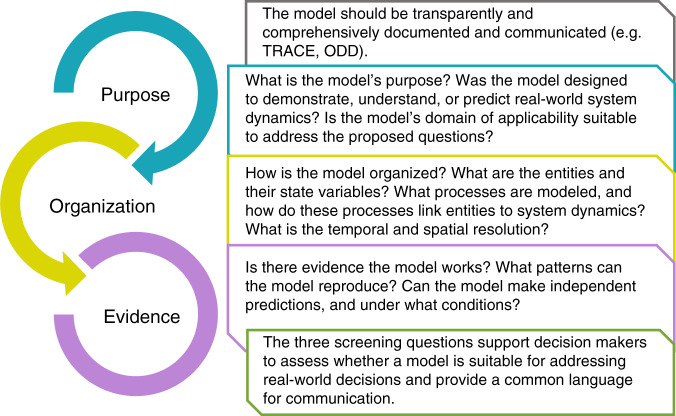
Three screening questions on a model’s purpose, organization, and evidence. They support the assessment of whether a model is suitable for addressing real-world decisions and how to use model results. They provide a common language for modelers and decision makers. They help to evaluate models and take them into account according to their purpose and evidence (TRACE: TRAnsparent and Comprehensive model “Evaludation”^8^); ODD: overview, design concepts, details^10^).

## Question 1: what is the model’s purpose?

Models are developed for a specific purpose and by the need to address certain questions about real systems. Models therefore focus on aspects of the real system that are considered important in answering these questions. Consequently, different models exist for the same system. Without knowing its purpose, it is impossible to assess whether a model’s outputs can be used to support decisions affecting the real world.

Model purposes fall into three main categories: demonstration, understanding, and prediction. Given these different purposes, models also reflect different scopes. Models for demonstration are designed to explore ideas, demonstrate the consequences of certain assumptions, and thereby help communicate key concepts and mechanisms. For example, at the onset of the Covid-19 pandemic simple mathematical models were used to demonstrate how lowering the basic reproduction value, *R*_0_, would lead to “flattening the curve” of infections over time. This is an important logical prediction that helped to make key decisions, but it does not, and cannot, say anything about how effective interventions like social distancing are in reducing *R*_0_.

Models for understanding are aimed at exploring how different components of a system interact to shape observed behavior of real systems. For example, a model can mechanistically represent movement and contact rates of individuals. The model can be run to let *R*_0_ emerge and then explore how *R*_0_ changes with interventions such as social distancing. Such models are not necessarily numerically precise, but they provide mechanistic understanding that helps to evaluate the consequences of alternative management measures.

Finally, models for prediction focus on numerical precision. They tend to be more detailed and complex and rely heavily on data for calibration. Their ability to make future projections therefore depends on the quality of data used for model calibration. Such models still do not predict the future with precision, as this is impossible^11^, but they provide important estimates of alternative future scenarios^12^.

Decision makers can benefit from all three types of models if they use them according to their given purpose. Modelers should therefore state a model’s purpose clearly and upfront. By asking this first screening question, one of the most common misuses of models can be prevented: using them for purposes for which they were not designed^13^.

## Question 2: how is the model organized?

Models are often used to support decisions without sufficient understanding of a model’s basic elements. Modelers need to ensure that their models are comprehensively documented, so that these elements can be understood by decision makers. The relevant questions about a model’s organization are: What is in the model, and how do the parts of the model work together (Fig. 1)?

Decision makers can quickly understand which aspects of the real world are included, and which are excluded, by assessing: what entities are present in the model (e.g., individuals, populations, companies), what state variables characterize these entities (e.g., age, nationality, bank balance), what processes (e.g., movement patterns, meeting rates) link entities and their variables to system dynamics, and what are the temporal and spatial resolution and extent?

Recognizing the entities, processes and scales of a model provides vital information about a model’s scope and hence its potential utility in a decision situation. For example, a model that does not include features of the economy cannot be used to explore the economic impact of lockdowns. Likewise, a model’s temporal resolution determines whether, for example, short-term behavioral changes can be considered. Taken together, information about a model’s structure, processes, and scales provides a quick overview of the key assumptions of the model, including the factors that the model ignores. These assumptions are then open to discussion, they can be compared, and their empirical basis can be checked. According to GMP, the rationale underlying these key assumptions should be documented^8^.

## Question 3: is there evidence the model works?

Real systems are characterized by features that persist over time and can be quantified. Models are designed and parameterized to reproduce one or more of these features, which can broadly be referred to as “patterns”. The patterns used for model design and parameterization, however, can be idiosyncratic. That is, experts often disagree about the important characteristics of a system and tend to focus on the patterns with which they are familiar. The only way to deal with this uncertainty is to be explicit about the patterns and data used and why they were considered important. Nevertheless, models need to replicate features of the system pertinent to answering the questions at hand, and therefore should be able to demonstrate that incorrect assumptions lead to poor replication of important patterns.

Models for demonstration focus on generic features such as the existence of an equilibrium, which confirms the modeled system exists. Models for understanding focus on one or a subset of mechanisms and how they help to replicate observed patterns. Models for prediction focus on quantitatively reproducing sets of patterns, observed at different scales and levels of the system’s organization. Each pattern is used as a filter to reject unsuitable parameter values or inaccurate representations of processes. The more patterns a model can reproduce simultaneously, for instance, by using a pattern-oriented modeling approach, the more reliably it captures the essential features of a real system’s organization^14^.

Patterns need not be highly distinctive. A combination of broad weak patterns can be as informative as a strong high-resolution pattern. We are all familiar with this power of weak but combined filters: we can pick up a person unknown to us at the airport if we have their photo (strong pattern), or if we are told the person is, e.g., male, wears glasses, and has a red suitcase (combination of weak patterns). For situations such as the Covid-19 pandemic, where few data and little knowledge are available at first, it is particularly important that models can predict multiple patterns, which by themselves may not contain much information but in combination can reduce model uncertainty.

In crisis situations, like in a pandemic or the conservation of threatened species, models are often limited by incomplete data. Nevertheless, models can help identify the most sensitive factors so that data collection can be prioritized. It is also essential that models can be readily updated with emerging information, which is facilitated by clear communication of a model’s organization.

## A way forward

Models tend to look right as they are presented with the claim of being realistic enough for their purpose. Looking right, though, has many dimensions, and we suggest the three screening questions presented here as a simple approach for decision makers to disentangle them. Once we know a model’s purpose, we can assess whether its organization is meaningful; once we know its purpose and organization, we can assess whether it represents the real system dynamics relevant to a specific problem; once we know which patterns it can reproduce, we can assess whether its model outputs can support decisions; and once we have answered all three questions for a suite of models they can be compared and taken into account to varying degrees^5^. The three questions do not replace more detailed guidelines on GMP^6,7^, but they provide a simple and effective common language that will allow us to develop models and use their outputs for decision support in a more transparent, robust, and safe way.

It is still important to keep in mind that models can only support decisions. The responsibility for using model outputs lies with decision makers. Answers to the three screening questions allow them to transparently base their decisions on a weight-of-evidence approach^15^, and to update their decisions when new data and updated models are available.
